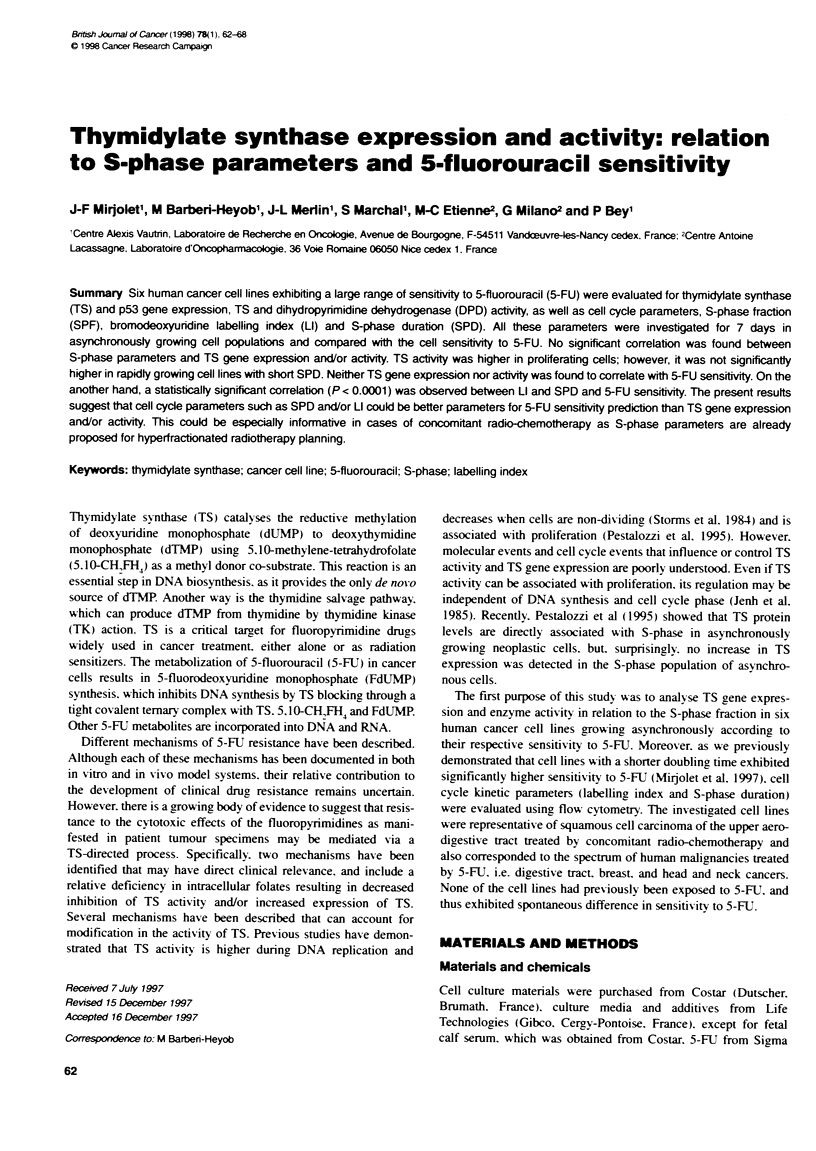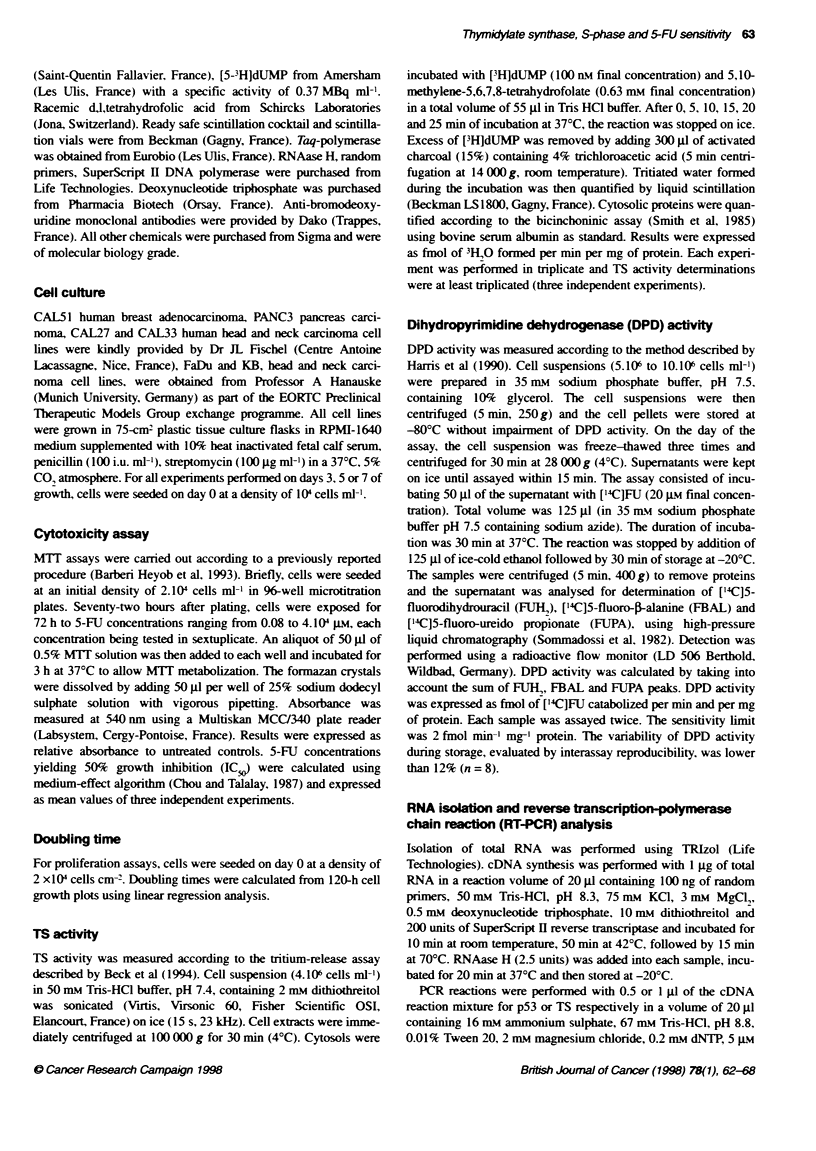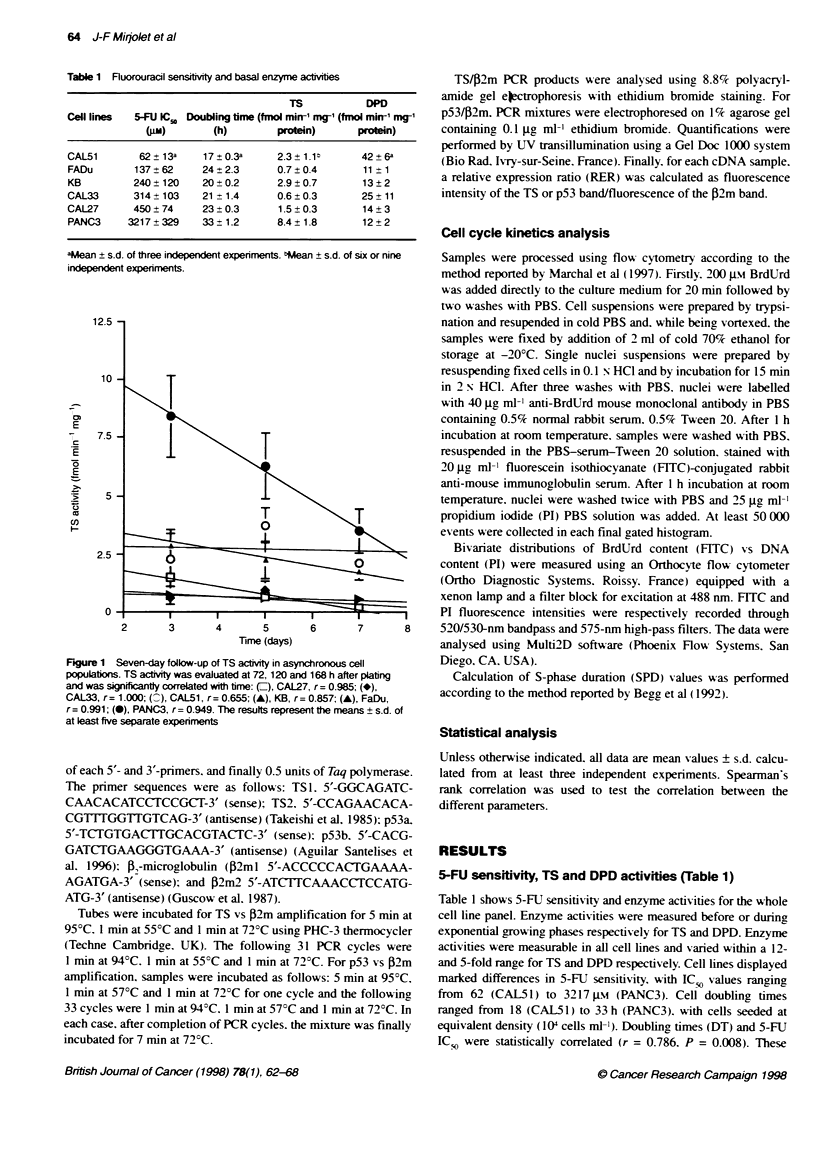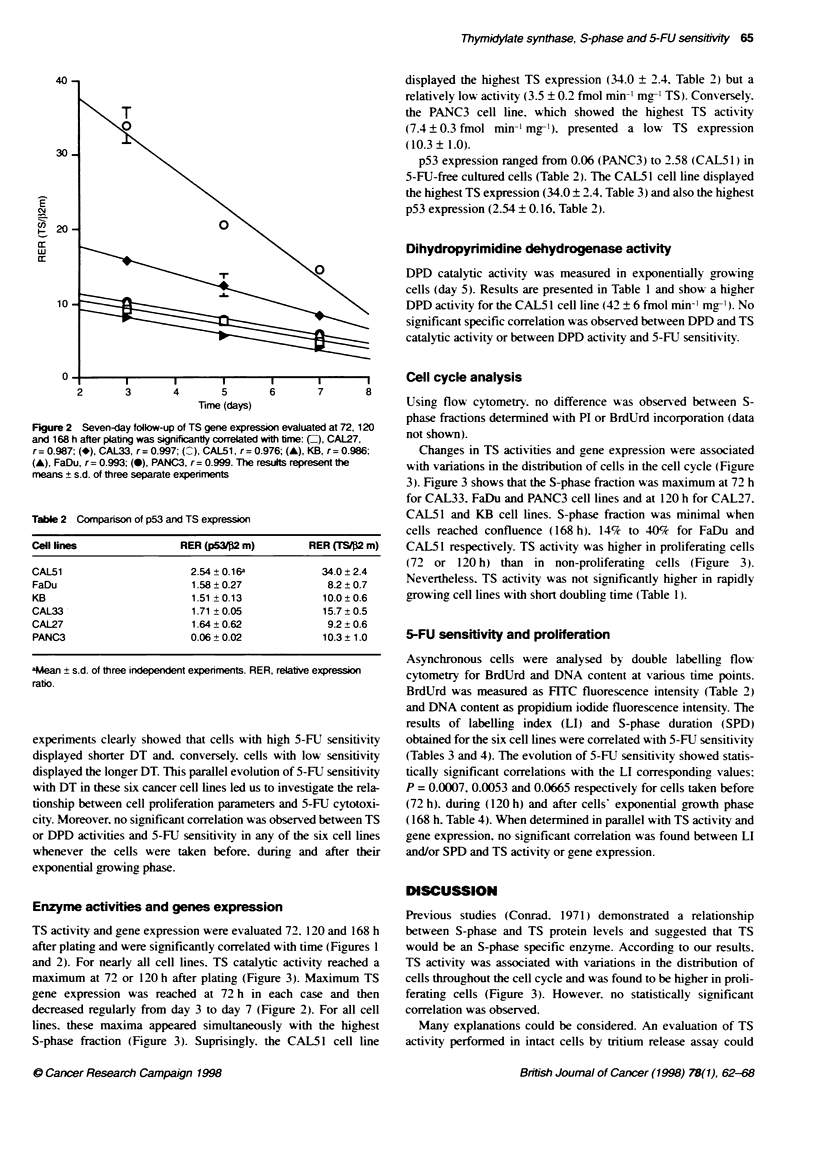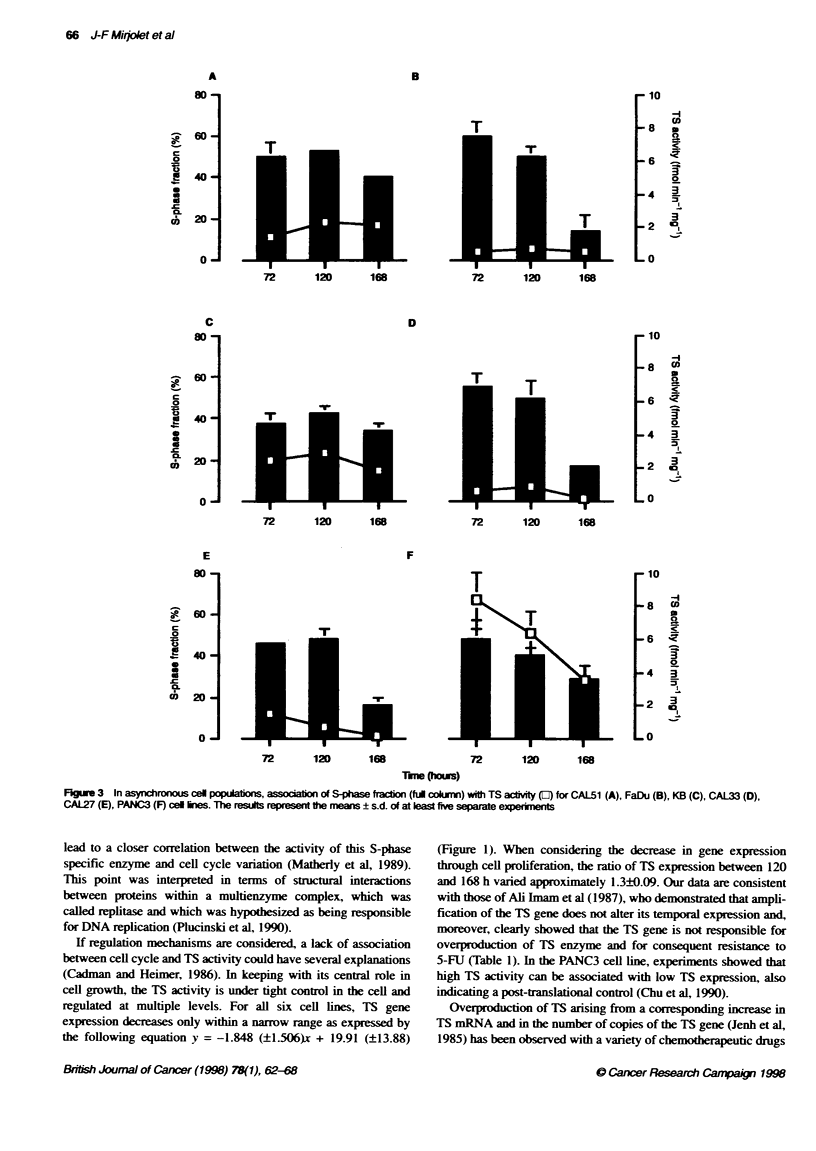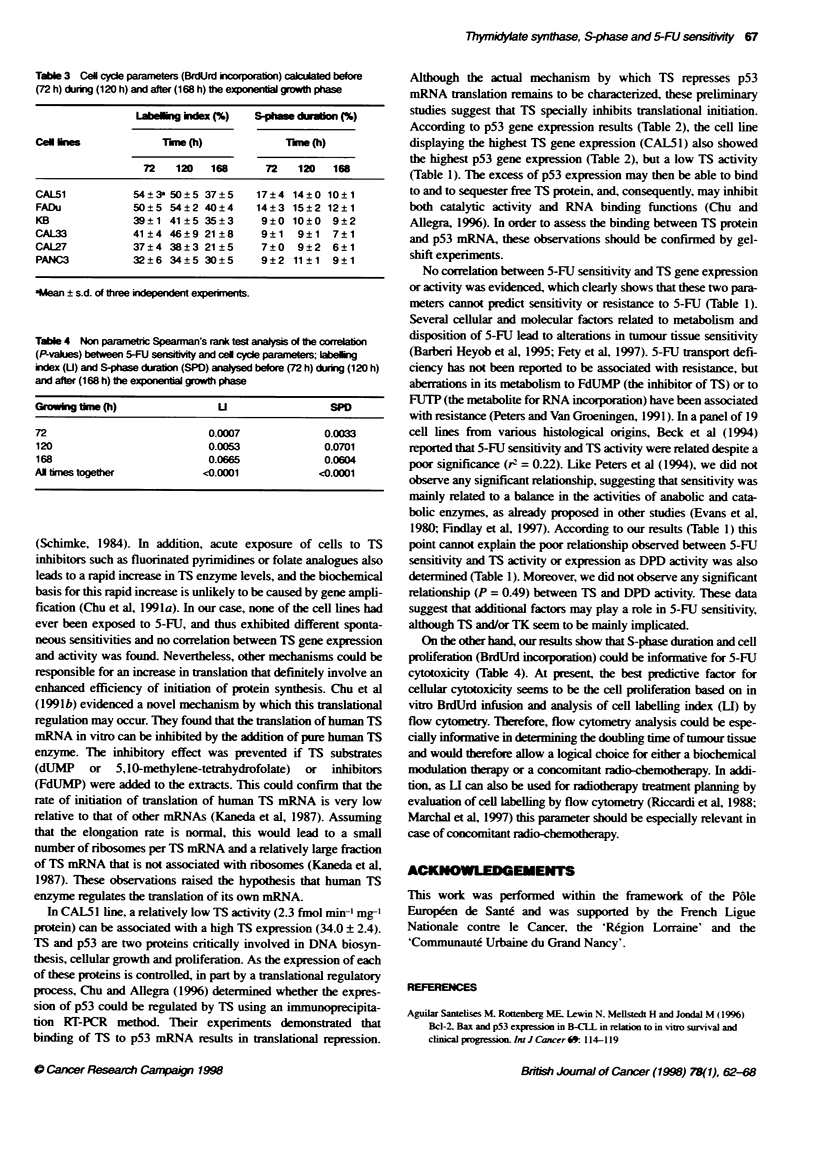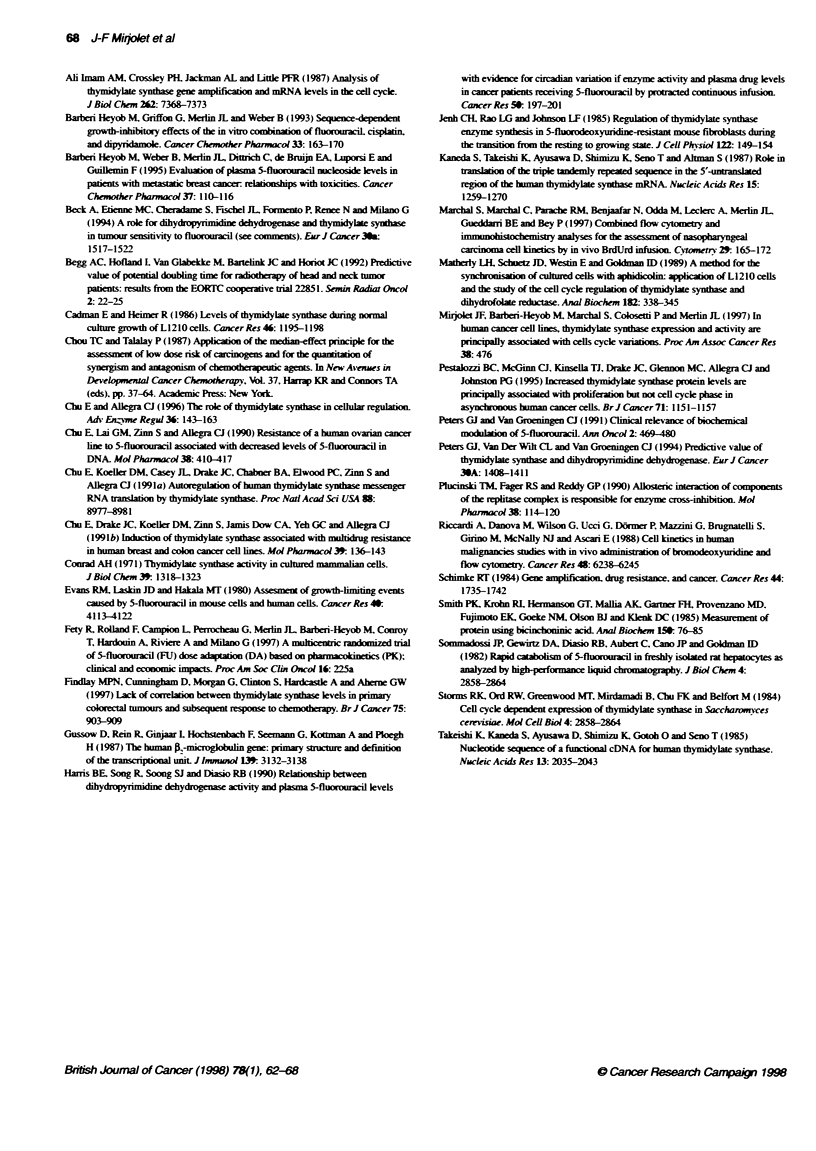# Thymidylate synthase expression and activity: relation to S-phase parameters and 5-fluorouracil sensitivity.

**DOI:** 10.1038/bjc.1998.443

**Published:** 1998-07

**Authors:** J. F. Mirjolet, M. Barberi-Heyob, J. L. Merlin, S. Marchal, M. C. Etienne, G. Milano, P. Bey

**Affiliations:** Centre Alexis Vautrin, Laboratoire de Recherche en Oncologie, Vandoeuvre-les-Nancy, France.

## Abstract

Six human cancer cell lines exhibiting a large range of sensitivity to 5-fluorouracil (5-FU) were evaluated for thymidylate synthase (TS) and p53 gene expression, TS and dihydropyrimidine dehydrogenase (DPD) activity, as well as cell cycle parameters, S-phase fraction (SPF), bromodeoxyuridine labelling index (LI) and S-phase duration (SPD). All these parameters were investigated for 7 days in asynchronously growing cell populations and compared with the cell sensitivity to 5-FU. No significant correlation was found between S-phase parameters and TS gene expression and/or activity. TS activity was higher in proliferating cells; however, it was not significantly higher in rapidly growing cell lines with short SPD. Neither TS gene expression nor activity was found to correlate with 5-FU sensitivity. On the another hand, a statistically significant correlation (P < 0.0001) was observed between LI and SPD and 5-FU sensitivity. The present results suggest that cell cycle parameters such as SPD and/or LI could be better parameters for 5-FU sensitivity prediction than TS gene expression and/or activity. This could be especially informative in cases of concomitant radio-chemotherapy as S-phase parameters are already proposed for hyperfractionated radiotherapy planning.


British Journal of Cancer (1998) 78(1), 62–68
© 1998 Cancer Research Campaign

### Thymidylate synthase expression and activity: relation to S-phase parameters and 5-fluorouracil sensitivity

J-F Mirjolet[1], M Barberi-Heyob[1], J-L Merlin[1], S Marchal[1], M-C Etienne[2], G Milano[2] and P Bey[1]

[1]Centre Alexis Vautrin, Laboratoire de Recherche en Oncologie, Avenue de Bourgogne, F-54511 Vandœuvre-les-Nancy cedex, France; [2]Centre Antoine Lacassagne, Laboratoire d'Oncopharmacologie, 36 Voie Romaine 06050 Nice cedex 1, France

**Summary** Six human cancer cell lines exhibiting a large range of sensitivity to 5-fluorouracil (5-FU) were evaluated for thymidylate synthase (TS) and p53 gene expression, TS and dihydropyrimidine dehydrogenase (DPD) activity, as well as cell cycle parameters, S-phase fraction (SPF), bromodeoxyuridine labelling index (LI) and S-phase duration (SPD). All these parameters were investigated for 7 days in asynchronously growing cell populations and compared with the cell sensitivity to 5-FU. No significant correlation was found between S-phase parameters and TS gene expression and/or activity. TS activity was higher in proliferating cells; however, it was not significantly higher in rapidly growing cell lines with short SPD. Neither TS gene expression nor activity was found to correlate with 5-FU sensitivity. On the another hand, a statistically significant correlation ($P < 0.0001$) was observed between LI and SPD and 5-FU sensitivity. The present results suggest that cell cycle parameters such as SPD and/or LI could be better parameters for 5-FU sensitivity prediction than TS gene expression and/or activity. This could be especially informative in cases of concomitant radio-chemotherapy as S-phase parameters are already proposed for hyperfractionated radiotherapy planning.

**Keywords:** thymidylate synthase; cancer cell line; 5-fluorouracil; S-phase; labelling index

Thymidylate synthase (TS) catalyses the reductive methylation of deoxyuridine monophosphate (dUMP) to deoxythymidine monophosphate (dTMP) using 5,10-methylene-tetrahydrofolate (5,10-$CH_2FH_4$) as a methyl donor co-substrate. This reaction is an essential step in DNA biosynthesis, as it provides the only *de novo* source of dTMP. Another way is the thymidine salvage pathway, which can produce dTMP from thymidine by thymidine kinase (TK) action. TS is a critical target for fluoropyrimidine drugs widely used in cancer treatment, either alone or as radiation sensitizers. The metabolization of 5-fluorouracil (5-FU) in cancer cells results in 5-fluorodeoxyuridine monophosphate (FdUMP) synthesis, which inhibits DNA synthesis by TS blocking through a tight covalent ternary complex with TS, 5,10-$CH_2FH_4$ and FdUMP. Other 5-FU metabolites are incorporated into DNA and RNA.

Different mechanisms of 5-FU resistance have been described. Although each of these mechanisms has been documented in both in vitro and in vivo model systems, their relative contribution to the development of clinical drug resistance remains uncertain. However, there is a growing body of evidence to suggest that resistance to the cytotoxic effects of the fluoropyrimidines as manifested in patient tumour specimens may be mediated via a TS-directed process. Specifically, two mechanisms have been identified that may have direct clinical relevance, and include a relative deficiency in intracellular folates resulting in decreased inhibition of TS activity and/or increased expression of TS. Several mechanisms have been described that can account for modification in the activity of TS. Previous studies have demonstrated that TS activity is higher during DNA replication and decreases when cells are non-dividing (Storms et al, 1984) and is associated with proliferation (Pestalozzi et al, 1995). However, molecular events and cell cycle events that influence or control TS activity and TS gene expression are poorly understood. Even if TS activity can be associated with proliferation, its regulation may be independent of DNA synthesis and cell cycle phase (Jenh et al, 1985). Recently, Pestalozzi et al (1995) showed that TS protein levels are directly associated with S-phase in asynchronously growing neoplastic cells, but, surprisingly, no increase in TS expression was detected in the S-phase population of asynchronous cells.

The first purpose of this study was to analyse TS gene expression and enzyme activity in relation to the S-phase fraction in six human cancer cell lines growing asynchronously according to their respective sensitivity to 5-FU. Moreover, as we previously demonstrated that cell lines with a shorter doubling time exhibited significantly higher sensitivity to 5-FU (Mirjolet et al, 1997), cell cycle kinetic parameters (labelling index and S-phase duration) were evaluated using flow cytometry. The investigated cell lines were representative of squamous cell carcinoma of the upper aerodigestive tract treated by concomitant radio-chemotherapy and also corresponded to the spectrum of human malignancies treated by 5-FU, i.e. digestive tract, breast, and head and neck cancers. None of the cell lines had previously been exposed to 5-FU, and thus exhibited spontaneous difference in sensitivity to 5-FU.

### MATERIALS AND METHODS

### Materials and chemicals

Cell culture materials were purchased from Costar (Dutscher, Brumath, France), culture media and additives from Life Technologies (Gibco, Cergy-Pontoise, France), except for fetal calf serum, which was obtained from Costar. 5-FU from Sigma

*Received 7 July 1997*
*Revised 15 December 1997*
*Accepted 16 December 1997*

*Correspondence to:* M Barberi-Heyob

(Saint-Quentin Fallavier, France), [5-$^3$H]dUMP from Amersham (Les Ulis, France) with a specific activity of 0.37 MBq ml$^{-1}$. Racemic d,l,tetrahydrofolic acid from Schircks Laboratories (Jona, Switzerland). Ready safe scintillation cocktail and scintillation vials were from Beckman (Gagny, France). *Taq*-polymerase was obtained from Eurobio (Les Ulis, France). RNAase H, random primers, SuperScript II DNA polymerase were purchased from Life Technologies. Deoxynucleotide triphosphate was purchased from Pharmacia Biotech (Orsay, France). Anti-bromodeoxyuridine monoclonal antibodies were provided by Dako (Trappes, France). All other chemicals were purchased from Sigma and were of molecular biology grade.

### Cell culture

CAL51 human breast adenocarcinoma, PANC3 pancreas carcinoma, CAL27 and CAL33 human head and neck carcinoma cell lines were kindly provided by Dr JL Fischel (Centre Antoine Lacassagne, Nice, France), FaDu and KB, head and neck carcinoma cell lines, were obtained from Professor A Hanauske (Munich University, Germany) as part of the EORTC Preclinical Therapeutic Models Group exchange programme. All cell lines were grown in 75-cm$^2$ plastic tissue culture flasks in RPMI-1640 medium supplemented with 10% heat inactivated fetal calf serum, penicillin (100 i.u. ml$^{-1}$), streptomycin (100 μg ml$^{-1}$) in a 37°C, 5% $CO_2$ atmosphere. For all experiments performed on days 3, 5 or 7 of growth, cells were seeded on day 0 at a density of 10$^4$ cells ml$^{-1}$.

### Cytotoxicity assay

MTT assays were carried out according to a previously reported procedure (Barberi Heyob et al, 1993). Briefly, cells were seeded at an initial density of 2.10$^4$ cells ml$^{-1}$ in 96-well microtitration plates. Seventy-two hours after plating, cells were exposed for 72 h to 5-FU concentrations ranging from 0.08 to 4.10$^4$ μM, each concentration being tested in sextuplicate. An aliquot of 50 μl of 0.5% MTT solution was then added to each well and incubated for 3 h at 37°C to allow MTT metabolization. The formazan crystals were dissolved by adding 50 μl per well of 25% sodium dodecyl sulphate solution with vigorous pipetting. Absorbance was measured at 540 nm using a Multiskan MCC/340 plate reader (Labsystem, Cergy-Pontoise, France). Results were expressed as relative absorbance to untreated controls. 5-FU concentrations yielding 50% growth inhibition ($IC_{50}$) were calculated using medium-effect algorithm (Chou and Talalay, 1987) and expressed as mean values of three independent experiments.

### Doubling time

For proliferation assays, cells were seeded on day 0 at a density of 2 ×10$^4$ cells cm$^{-2}$. Doubling times were calculated from 120-h cell growth plots using linear regression analysis.

### TS activity

TS activity was measured according to the tritium-release assay described by Beck et al (1994). Cell suspension (4.10$^6$ cells ml$^{-1}$) in 50 mM Tris-HCl buffer, pH 7.4, containing 2 mM dithiothreitol was sonicated (Virtis, Virsonic 60, Fisher Scientific OSI, Elancourt, France) on ice (15 s, 23 kHz). Cell extracts were immediately centrifuged at 100 000 *g* for 30 min (4°C). Cytosols were incubated with [$^3$H]dUMP (100 nM final concentration) and 5,10-methylene-5,6,7,8-tetrahydrofolate (0.63 mM final concentration) in a total volume of 55 μl in Tris HCl buffer. After 0, 5, 10, 15, 20 and 25 min of incubation at 37°C, the reaction was stopped on ice. Excess of [$^3$H]dUMP was removed by adding 300 μl of activated charcoal (15%) containing 4% trichloroacetic acid (5 min centrifugation at 14 000 *g*, room temperature). Tritiated water formed during the incubation was then quantified by liquid scintillation (Beckman LS1800, Gagny, France). Cytosolic proteins were quantified according to the bicinchoninic assay (Smith et al, 1985) using bovine serum albumin as standard. Results were expressed as fmol of $^3H_2O$ formed per min per mg of protein. Each experiment was performed in triplicate and TS activity determinations were at least triplicated (three independent experiments).

### Dihydropyrimidine dehydrogenase (DPD) activity

DPD activity was measured according to the method described by Harris et al (1990). Cell suspensions (5.10$^6$ to 10.10$^6$ cells ml$^{-1}$) were prepared in 35 mM sodium phosphate buffer, pH 7.5, containing 10% glycerol. The cell suspensions were then centrifuged (5 min, 250 *g*) and the cell pellets were stored at –80°C without impairment of DPD activity. On the day of the assay, the cell suspension was freeze–thawed three times and centrifuged for 30 min at 28 000 *g* (4°C). Supernatants were kept on ice until assayed within 15 min. The assay consisted of incubating 50 μl of the supernatant with [$^{14}$C]FU (20 μM final concentration). Total volume was 125 μl (in 35 mM sodium phosphate buffer pH 7.5 containing sodium azide). The duration of incubation was 30 min at 37°C. The reaction was stopped by addition of 125 μl of ice-cold ethanol followed by 30 min of storage at –20°C. The samples were centrifuged (5 min, 400 *g*) to remove proteins and the supernatant was analysed for determination of [$^{14}$C]5-fluorodihydrouracil ($FUH_2$), [$^{14}$C]5-fluoro-β-alanine (FBAL) and [$^{14}$C]5-fluoro-ureido propionate (FUPA), using high-pressure liquid chromatography (Sommadossi et al, 1982). Detection was performed using a radioactive flow monitor (LD 506 Berthold, Wildbad, Germany). DPD activity was calculated by taking into account the sum of $FUH_2$, FBAL and FUPA peaks. DPD activity was expressed as fmol of [$^{14}$C]FU catabolized per min and per mg of protein. Each sample was assayed twice. The sensitivity limit was 2 fmol min$^{-1}$ mg$^{-1}$ protein. The variability of DPD activity during storage, evaluated by interassay reproducibility, was lower than 12% ($n$ = 8).

### RNA isolation and reverse transcription-polymerase chain reaction (RT-PCR) analysis

Isolation of total RNA was performed using TRIzol (Life Technologies). cDNA synthesis was performed with 1 μg of total RNA in a reaction volume of 20 μl containing 100 ng of random primers, 50 mM Tris-HCl, pH 8.3, 75 mM KCl, 3 mM $MgCl_2$, 0.5 mM deoxynucleotide triphosphate, 10 mM dithiothreitol and 200 units of SuperScript II reverse transcriptase and incubated for 10 min at room temperature, 50 min at 42°C, followed by 15 min at 70°C. RNAase H (2.5 units) was added into each sample, incubated for 20 min at 37°C and then stored at –20°C.

PCR reactions were performed with 0.5 or 1 μl of the cDNA reaction mixture for p53 or TS respectively in a volume of 20 μl containing 16 mM ammonium sulphate, 67 mM Tris-HCl, pH 8.8, 0.01% Tween 20, 2 mM magnesium chloride, 0.2 mM dNTP, 5 μM

© Cancer Research Campaign 1998 

**Table 1** Fluorouracil sensitivity and basal enzyme activities

| Cell lines | 5-FU $IC_{50}$ (μM) | Doubling time (h) | TS (fmol $min^{-1}$ $mg^{-1}$ protein) | DPD (fmol $min^{-1}$ $mg^{-1}$ protein) |
|---|---|---|---|---|
| CAL51 | 62 ± 13[a] | 17 ± 0.3[a] | 2.3 ± 1.1[b] | 42 ± 6[a] |
| FADu | 137 ± 62 | 24 ± 2.3 | 0.7 ± 0.4 | 11 ± 1 |
| KB | 240 ± 120 | 20 ± 0.2 | 2.9 ± 0.7 | 13 ± 2 |
| CAL33 | 314 ± 103 | 21 ± 1.4 | 0.6 ± 0.3 | 25 ± 11 |
| CAL27 | 450 ± 74 | 23 ± 0.3 | 1.5 ± 0.3 | 14 ± 3 |
| PANC3 | 3217 ± 329 | 33 ± 1.2 | 8.4 ± 1.8 | 12 ± 2 |

[a]Mean ± s.d. of three independent experiments. [b]Mean ± s.d. of six or nine independent experiments.

**Figure 1** Seven-day follow-up of TS activity in asynchronous cell populations. TS activity was evaluated at 72, 120 and 168 h after plating and was significantly correlated with time: (□), CAL27, $r$ = 0.985; (◆), CAL33, $r$ = 1.000; (○), CAL51, $r$ = 0.655; (▲), KB, $r$ = 0.857; (▲), FaDu, $r$ = 0.991; (●), PANC3, $r$ = 0.949. The results represent the means ± s.d. of at least five separate experiments of each 5′- and 3′-primers, and finally 0.5 units of *Taq* polymerase. The primer sequences were as follows: TS1, 5′-GGCAGATC-CAACACATCCTCCGCT-3′ (sense); TS2, 5′-CCAGAACACA-CGTTTGGTTGTCAG-3′ (antisense) (Takeishi et al, 1985); p53a, 5′-TCTGTGACTTGCACGTACTC-3′ (sense); p53b, 5′-CACG-GATCTGAAGGGTGAAA-3′ (antisense) (Aguilar Santelises et al, 1996); $\beta_2$-microglobulin (β2m1 5′-ACCCCCACTGAAAA-AGATGA-3′ (sense); and β2m2 5′-ATCTTCAAACCTCCATG-ATG-3′ (antisense) (Guscow et al, 1987).

Tubes were incubated for TS vs β2m amplification for 5 min at 95°C, 1 min at 55°C and 1 min at 72°C using PHC-3 thermocycler (Techne Cambridge, UK). The following 31 PCR cycles were 1 min at 94°C, 1 min at 55°C and 1 min at 72°C. For p53 vs β2m amplification, samples were incubated as follows: 5 min at 95°C, 1 min at 57°C and 1 min at 72°C for one cycle and the following 33 cycles were 1 min at 94°C, 1 min at 57°C and 1 min at 72°C. In each case, after completion of PCR cycles, the mixture was finally incubated for 7 min at 72°C.

TS/β2m PCR products were analysed using 8.8% polyacrylamide gel electrophoresis with ethidium bromide staining. For p53/β2m, PCR mixtures were electrophoresed on 1% agarose gel containing 0.1 μg $ml^{-1}$ ethidium bromide. Quantifications were performed by UV transillumination using a Gel Doc 1000 system (Bio Rad, Ivry-sur-Seine, France). Finally, for each cDNA sample, a relative expression ratio (RER) was calculated as fluorescence intensity of the TS or p53 band/fluorescence of the β2m band.

### Cell cycle kinetics analysis

Samples were processed using flow cytometry according to the method reported by Marchal et al (1997). Firstly, 200 μM BrdUrd was added directly to the culture medium for 20 min followed by two washes with PBS. Cell suspensions were prepared by trypsination and resupended in cold PBS and, while being vortexed, the samples were fixed by addition of 2 ml of cold 70% ethanol for storage at −20°C. Single nuclei suspensions were prepared by resuspending fixed cells in 0.1 N HCl and by incubation for 15 min in 2 N HCl. After three washes with PBS, nuclei were labelled with 40 μg $ml^{-1}$ anti-BrdUrd mouse monoclonal antibody in PBS containing 0.5% normal rabbit serum, 0.5% Tween 20. After 1 h incubation at room temperature, samples were washed with PBS, resuspended in the PBS–serum–Tween 20 solution, stained with 20 μg $ml^{-1}$ fluorescein isothiocyanate (FITC)-conjugated rabbit anti-mouse immunoglobulin serum. After 1 h incubation at room temperature, nuclei were washed twice with PBS and 25 μg $ml^{-1}$ propidium iodide (PI) PBS solution was added. At least 50 000 events were collected in each final gated histogram.

Bivariate distributions of BrdUrd content (FITC) vs DNA content (PI) were measured using an Orthocyte flow cytometer (Ortho Diagnostic Systems, Roissy, France) equipped with a xenon lamp and a filter block for excitation at 488 nm. FITC and PI fluorescence intensities were respectively recorded through 520/530-nm bandpass and 575-nm high-pass filters. The data were analysed using Multi2D software (Phoenix Flow Systems, San Diego, CA, USA).

Calculation of S-phase duration (SPD) values was performed according to the method reported by Begg et al (1992).

### Statistical analysis

Unless otherwise indicated, all data are mean values ± s.d. calculated from at least three independent experiments. Spearman's rank correlation was used to test the correlation between the different parameters.

### RESULTS

### 5-FU sensitivity, TS and DPD activities (Table 1)

Table 1 shows 5-FU sensitivity and enzyme activities for the whole cell line panel. Enzyme activities were measured before or during exponential growing phases respectively for TS and DPD. Enzyme activities were measurable in all cell lines and varied within a 12- and 5-fold range for TS and DPD respectively. Cell lines displayed marked differences in 5-FU sensitivity, with $IC_{50}$ values ranging from 62 (CAL51) to 3217 μM (PANC3). Cell doubling times ranged from 18 (CAL51) to 33 h (PANC3), with cells seeded at equivalent density ($10^4$ cells $ml^{-1}$). Doubling times (DT) and 5-FU $IC_{50}$ were statistically correlated ($r$ = 0.786, $P$ = 0.008). These

© Cancer Research Campaign 1998

**Figure 2** Seven-day follow-up of TS gene expression evaluated at 72, 120 and 168 h after plating was significantly correlated with time: (□), CAL27, $r$ = 0.987; (◆), CAL33, $r$ = 0.997; (○), CAL51, $r$ = 0.976; (▲), KB, $r$ = 0.986; (▲), FaDu, $r$ = 0.993; (●), PANC3, $r$ = 0.999. The results represent the means ± s.d. of three separate experiments

**Table 2** Comparison of p53 and TS expression

| Cell lines | RER (p53/β2 m) | RER (TS/β2 m) |
|---|---|---|
| CAL51 | 2.54 ± 0.16[a] | 34.0 ± 2.4 |
| FaDu | 1.58 ± 0.27 | 8.2 ± 0.7 |
| KB | 1.51 ± 0.13 | 10.0 ± 0.6 |
| CAL33 | 1.71 ± 0.05 | 15.7 ± 0.5 |
| CAL27 | 1.64 ± 0.62 | 9.2 ± 0.6 |
| PANC3 | 0.06 ± 0.02 | 10.3 ± 1.0 |

[a]Mean ± s.d. of three independent experiments. RER, relative expression ratio.

experiments clearly showed that cells with high 5-FU sensitivity displayed shorter DT and, conversely, cells with low sensitivity displayed the longer DT. This parallel evolution of 5-FU sensitivity with DT in these six cancer cell lines led us to investigate the relationship between cell proliferation parameters and 5-FU cytotoxicity. Moreover, no significant correlation was observed between TS or DPD activities and 5-FU sensitivity in any of the six cell lines whenever the cells were taken before, during and after their exponential growing phase.

### Enzyme activities and genes expression

TS activity and gene expression were evaluated 72, 120 and 168 h after plating and were significantly correlated with time (Figures 1 and 2). For nearly all cell lines, TS catalytic activity reached a maximum at 72 or 120 h after plating (Figure 3). Maximum TS gene expression was reached at 72 h in each case and then decreased regularly from day 3 to day 7 (Figure 2). For all cell lines, these maxima appeared simultaneously with the highest S-phase fraction (Figure 3). Suprisingly, the CAL51 cell line displayed the highest TS expression (34.0 ± 2.4, Table 2) but a relatively low activity (3.5 ± 0.2 fmol $min^{-1}$ $mg^{-1}$ TS). Conversely, the PANC3 cell line, which showed the highest TS activity (7.4 ± 0.3 fmol $min^{-1}$ $mg^{-1}$), presented a low TS expression (10.3 ± 1.0).

p53 expression ranged from 0.06 (PANC3) to 2.58 (CAL51) in 5-FU-free cultured cells (Table 2). The CAL51 cell line displayed the highest TS expression (34.0 ± 2.4, Table 3) and also the highest p53 expression (2.54 ± 0.16, Table 2).

### Dihydropyrimidine dehydrogenase activity

DPD catalytic activity was measured in exponentially growing cells (day 5). Results are presented in Table 1 and show a higher DPD activity for the CAL51 cell line (42 ± 6 fmol $min^{-1}$ $mg^{-1}$). No significant specific correlation was observed between DPD and TS catalytic activity or between DPD activity and 5-FU sensitivity.

### Cell cycle analysis

Using flow cytometry, no difference was observed between S-phase fractions determined with PI or BrdUrd incorporation (data not shown).

Changes in TS activities and gene expression were associated with variations in the distribution of cells in the cell cycle (Figure 3). Figure 3 shows that the S-phase fraction was maximum at 72 h for CAL33, FaDu and PANC3 cell lines and at 120 h for CAL27, CAL51 and KB cell lines. S-phase fraction was minimal when cells reached confluence (168 h), 14% to 40% for FaDu and CAL51 respectively. TS activity was higher in proliferating cells (72 or 120 h) than in non-proliferating cells (Figure 3). Nevertheless, TS activity was not significantly higher in rapidly growing cell lines with short doubling time (Table 1).

### 5-FU sensitivity and proliferation

Asynchronous cells were analysed by double labelling flow cytometry for BrdUrd and DNA content at various time points. BrdUrd was measured as FITC fluorescence intensity (Table 2) and DNA content as propidium iodide fluorescence intensity. The results of labelling index (LI) and S-phase duration (SPD) obtained for the six cell lines were correlated with 5-FU sensitivity (Tables 3 and 4). The evolution of 5-FU sensitivity showed statistically significant correlations with the LI corresponding values; $P$ = 0.0007, 0.0053 and 0.0665 respectively for cells taken before (72 h), during (120 h) and after cells' exponential growth phase (168 h, Table 4). When determined in parallel with TS activity and gene expression, no significant correlation was found between LI and/or SPD and TS activity or gene expression.

### DISCUSSION

Previous studies (Conrad, 1971) demonstrated a relationship between S-phase and TS protein levels and suggested that TS would be an S-phase specific enzyme. According to our results, TS activity was associated with variations in the distribution of cells throughout the cell cycle and was found to be higher in proliferating cells (Figure 3). However, no statistically significant correlation was observed.

Many explanations could be considered. An evaluation of TS activity performed in intact cells by tritium release assay could

© Cancer Research Campaign 1998 

**Figure 3** In asynchronous cell populations, association of S-phase fraction (full column) with TS activity (□) for CAL51 (A), FaDu (B), KB (C), CAL33 (D), CAL27 (E), PANC3 (F) cell lines. The results represent the means ± s.d. of at least five separate experiments lead to a closer correlation between the activity of this S-phase specific enzyme and cell cycle variation (Matherly et al, 1989). This point was interpreted in terms of structural interactions between proteins within a multienzyme complex, which was called replitase and which was hypothesized as being responsible for DNA replication (Plucinski et al, 1990).

If regulation mechanisms are considered, a lack of association between cell cycle and TS activity could have several explanations (Cadman and Heimer, 1986). In keeping with its central role in cell growth, the TS activity is under tight control in the cell and regulated at multiple levels. For all six cell lines, TS gene expression decreases only within a narrow range as expressed by the following equation $y = -1.848\ (\pm 1.506)x + 19.91\ (\pm 13.88)$ (Figure 1). When considering the decrease in gene expression through cell proliferation, the ratio of TS expression between 120 and 168 h varied approximately 1.3±0.09. Our data are consistent with those of Ali Imam et al (1987), who demonstrated that amplification of the TS gene does not alter its temporal expression and, moreover, clearly showed that the TS gene is not responsible for overproduction of TS enzyme and for consequent resistance to 5-FU (Table 1). In the PANC3 cell line, experiments showed that high TS activity can be associated with low TS expression, also indicating a post-translational control (Chu et al, 1990).

Overproduction of TS arising from a corresponding increase in TS mRNA and in the number of copies of the TS gene (Jenh et al, 1985) has been observed with a variety of chemotherapeutic drugs

© Cancer Research Campaign 1998

**Table 3** Cell cycle parameters (BrdUrd incorporation) calculated before (72 h) during (120 h) and after (168 h) the exponential growth phase

| Cell lines | Labelling index (%) Time (h) | | | S-phase duration (%) Time (h) | | |
|---|---|---|---|---|---|---|
| | 72 | 120 | 168 | 72 | 120 | 168 |
| CAL51 | 54 ± 3[a] | 50 ± 5 | 37 ± 5 | 17 ± 4 | 14 ± 0 | 10 ± 1 |
| FADu | 50 ± 5 | 54 ± 2 | 40 ± 4 | 14 ± 3 | 15 ± 2 | 12 ± 1 |
| KB | 39 ± 1 | 41 ± 5 | 35 ± 3 | 9 ± 0 | 10 ± 0 | 9 ± 2 |
| CAL33 | 41 ± 4 | 46 ± 9 | 21 ± 8 | 9 ± 1 | 9 ± 1 | 7 ± 1 |
| CAL27 | 37 ± 4 | 38 ± 3 | 21 ± 5 | 7 ± 0 | 9 ± 2 | 6 ± 1 |
| PANC3 | 32 ± 6 | 34 ± 5 | 30 ± 5 | 9 ± 2 | 11 ± 1 | 9 ± 1 |

[a]Mean ± s.d. of three independent experiments.

**Table 4** Non parametric Spearman's rank test analysis of the correlation (*P*-values) between 5-FU sensitivity and cell cycle parameters; labelling index (LI) and S-phase duration (SPD) analysed before (72 h) during (120 h) and after (168 h) the exponential growth phase

| Growing time (h) | LI | SPD |
|---|---|---|
| 72 | 0.0007 | 0.0033 |
| 120 | 0.0053 | 0.0701 |
| 168 | 0.0665 | 0.0604 |
| All times together | <0.0001 | <0.0001 |

(Schimke, 1984). In addition, acute exposure of cells to TS inhibitors such as fluorinated pyrimidines or folate analogues also leads to a rapid increase in TS enzyme levels, and the biochemical basis for this rapid increase is unlikely to be caused by gene amplification (Chu et al, 1991*a*). In our case, none of the cell lines had ever been exposed to 5-FU, and thus exhibited different spontaneous sensitivities and no correlation between TS gene expression and activity was found. Nevertheless, other mechanisms could be responsible for an increase in translation that definitely involve an enhanced efficiency of initiation of protein synthesis. Chu et al (1991*b*) evidenced a novel mechanism by which this translational regulation may occur. They found that the translation of human TS mRNA in vitro can be inhibited by the addition of pure human TS enzyme. The inhibitory effect was prevented if TS substrates (dUMP or 5,10-methylene-tetrahydrofolate) or inhibitors (FdUMP) were added to the extracts. This could confirm that the rate of initiation of translation of human TS mRNA is very low relative to that of other mRNAs (Kaneda et al, 1987). Assuming that the elongation rate is normal, this would lead to a small number of ribosomes per TS mRNA and a relatively large fraction of TS mRNA that is not associated with ribosomes (Kaneda et al, 1987). These observations raised the hypothesis that human TS enzyme regulates the translation of its own mRNA.

In CAL51 line, a relatively low TS activity (2.3 fmol $min^{-1}$ $mg^{-1}$ protein) can be associated with a high TS expression (34.0 ± 2.4). TS and p53 are two proteins critically involved in DNA biosynthesis, cellular growth and proliferation. As the expression of each of these proteins is controlled, in part by a translational regulatory process, Chu and Allegra (1996) determined whether the expression of p53 could be regulated by TS using an immunoprecipitation RT-PCR method. Their experiments demonstrated that binding of TS to p53 mRNA results in translational repression. Although the actual mechanism by which TS represses p53 mRNA translation remains to be characterized, these preliminary studies suggest that TS specially inhibits translational initiation. According to p53 gene expression results (Table 2), the cell line displaying the highest TS gene expression (CAL51) also showed the highest p53 gene expression (Table 2), but a low TS activity (Table 1). The excess of p53 expression may then be able to bind to and to sequester free TS protein, and, consequently, may inhibit both catalytic activity and RNA binding functions (Chu and Allegra, 1996). In order to assess the binding between TS protein and p53 mRNA, these observations should be confirmed by gel-shift experiments.

No correlation between 5-FU sensitivity and TS gene expression or activity was evidenced, which clearly shows that these two parameters cannot predict sensitivity or resistance to 5-FU (Table 1). Several cellular and molecular factors related to metabolism and disposition of 5-FU lead to alterations in tumour tissue sensitivity (Barberi Heyob et al, 1995; Fety et al, 1997). 5-FU transport deficiency has not been reported to be associated with resistance, but aberrations in its metabolism to FdUMP (the inhibitor of TS) or to FUTP (the metabolite for RNA incorporation) have been associated with resistance (Peters and Van Groeningen, 1991). In a panel of 19 cell lines from various histological origins, Beck et al (1994) reported that 5-FU sensitivity and TS activity were related despite a poor significance ($r^2$ = 0.22). Like Peters et al (1994), we did not observe any significant relationship, suggesting that sensitivity was mainly related to a balance in the activities of anabolic and catabolic enzymes, as already proposed in other studies (Evans et al, 1980; Findlay et al, 1997). According to our results (Table 1) this point cannot explain the poor relationship observed between 5-FU sensitivity and TS activity or expression as DPD activity was also determined (Table 1). Moreover, we did not observe any significant relationship ($P$ = 0.49) between TS and DPD activity. These data suggest that additional factors may play a role in 5-FU sensitivity, although TS and/or TK seem to be mainly implicated.

On the other hand, our results show that S-phase duration and cell proliferation (BrdUrd incorporation) could be informative for 5-FU cytotoxicity (Table 4). At present, the best predictive factor for cellular cytotoxicity seems to be the cell proliferation based on in vitro BrdUrd infusion and analysis of cell labelling index (LI) by flow cytometry. Therefore, flow cytometry analysis could be especially informative in determining the doubling time of tumour tissue and would therefore allow a logical choice for either a biochemical modulation therapy or a concomitant radio-chemotherapy. In addition, as LI can also be used for radiotherapy treatment planning by evaluation of cell labelling by flow cytometry (Riccardi et al, 1988; Marchal et al, 1997) this parameter should be especially relevant in case of concomitant radio-chemotherapy.

### ACKNOWLEDGEMENTS

This work was performed within the framework of the Pôle Européen de Santé and was supported by the French Ligue Nationale contre le Cancer, the 'Région Lorraine' and the 'Communauté Urbaine du Grand Nancy'.

### REFERENCES

Aguilar Santelises M, Rottenberg ME, Lewin N, Mellstedt H and Jondal M (1996) Bcl-2, Bax and p53 expression in B-CLL in relation to in vitro survival and clinical progression. *Int J Cancer* **69**: 114–119

© Cancer Research Campaign 1998 

Ali Imam AM, Crossley PH, Jackman AL and Little PFR (1987) Analysis of thymidylate synthase gene amplification and mRNA levels in the cell cycle. *J Biol Chem* **262**: 7368–7373

Barberi Heyob M, Griffon G, Merlin JL and Weber B (1993) Sequence-dependent growth-inhibitory effects of the in vitro combination of fluorouracil, cisplatin, and dipyridamole. *Cancer Chemother Pharmacol* **33**: 163–170

Barberi Heyob M, Weber B, Merlin JL, Dittrich C, de Bruijn EA, Luporsi E and Guillemin F (1995) Evaluation of plasma 5-fluorouracil nucleoside levels in patients with metastatic breast cancer: relationships with toxicities. *Cancer Chemother Pharmacol* **37**: 110–116

Beck A, Etienne MC, Cheradame S, Fischel JL, Formento P, Renee N and Milano G (1994) A role for dihydropyrimidine dehydrogenase and thymidylate synthase in tumour sensitivity to fluorouracil (see comments). *Eur J Cancer* **30a**: 1517–1522

Begg AC, Hofland I, Van Glabekke M, Bartelink JC and Horiot JC (1992) Predictive value of potential doubling time for radiotherapy of head and neck tumor patients: results from the EORTC cooperative trial 22851. *Semin Radiat Oncol* **2**: 22–25

Cadman E and Heimer R (1986) Levels of thymidylate synthase during normal culture growth of L1210 cells. *Cancer Res* **46**: 1195–1198

Chou TC and Talalay P (1987) Application of the median-effect principle for the assessment of low dose risk of carcinogens and for the quantitation of synergism and antagonism of chemotherapeutic agents. In *New Avenues in Developmental Cancer Chemotherapy*, Vol. 37, Harrap KR and Connors TA (eds), pp. 37–64. Academic Press: New York.

Chu E and Allegra CJ (1996) The role of thymidylate synthase in cellular regulation. *Adv Enzyme Regul* **36**: 143–163

Chu E, Lai GM, Zinn S and Allegra CJ (1990) Resistance of a human ovarian cancer line to 5-fluorouracil associated with decreased levels of 5-fluorouracil in DNA. *Mol Pharmacol* **38**: 410–417

Chu E, Koeller DM, Casey JL, Drake JC, Chabner BA, Elwood PC, Zinn S and Allegra CJ (1991*a*) Autoregulation of human thymidylate synthase messenger RNA translation by thymidylate synthase. *Proc Natl Acad Sci USA* **88**: 8977–8981

Chu E, Drake JC, Koeller DM, Zinn S, Jamis Dow CA, Yeh GC and Allegra CJ (1991*b*) Induction of thymidylate synthase associated with multidrug resistance in human breast and colon cancer cell lines. *Mol Pharmacol* **39**: 136–143

Conrad AH (1971) Thymidylate synthase activity in cultured mammalian cells. *J Biol Chem* **39**: 1318–1323

Evans RM, Laskin JD and Hakala MT (1980) Assesment of growth-limiting events caused by 5-fluorouracil in mouse cells and human cells. *Cancer Res* **40**: 4113–4122

Fety R, Rolland F, Campion L, Perrocheau G, Merlin JL, Barberi-Heyob M, Conroy T, Hardouin A, Riviere A and Milano G (1997) A multicentric randomized trial of 5-fluorouracil (FU) dose adaptation (DA) based on pharmacokinetics (PK): clinical and economic impacts. *Proc Am Soc Clin Oncol* **16**: 225a

Findlay MPN, Cunningham D, Morgan G, Clinton S, Hardcastle A and Aherne GW (1997) Lack of correlation between thymidylate synthase levels in primary colorectal tumours and subsequent response to chemotherapy. *Br J Cancer* **75**: 903–909

Gussow D, Rein R, Ginjaar I, Hochstenbach F, Seemann G, Kottman A and Ploegh H (1987) The human $\beta_2$-microglobulin gene: primary structure and definition of the transcriptional unit. *J Immunol* **139**: 3132–3138

Harris BE, Song R, Soong SJ and Diasio RB (1990) Relationship between dihydropyrimidine dehydrogenase activity and plasma 5-fluorouracil levels with evidence for circadian variation if enzyme activity and plasma drug levels in cancer patients receiving 5-fluorouracil by protracted continuous infusion. *Cancer Res* **50**: 197–201

Jenh CH, Rao LG and Johnson LF (1985) Regulation of thymidylate synthase enzyme synthesis in 5-fluorodeoxyuridine-resistant mouse fibroblasts during the transition from the resting to growing state. *J Cell Physiol* **122**: 149–154

Kaneda S, Takeishi K, Ayusawa D, Shimizu K, Seno T and Altman S (1987) Role in translation of the triple tandemly repeated sequence in the 5′-untranslated region of the human thymidylate synthase mRNA. *Nucleic Acids Res* **15**: 1259–1270

Marchal S, Marchal C, Parache RM, Benjaafar N, Odda M, Leclerc A, Merlin JL, Gueddarri BE and Bey P (1997) Combined flow cytometry and immunohistochemistry analyses for the assessment of nasopharyngeal carcinoma cell kinetics by in vivo BrdUrd infusion. *Cytometry* **29**: 165–172

Matherly LH, Schuetz JD, Westin E and Goldman ID (1989) A method for the synchronisation of cultured cells with aphidicolin: application of L1210 cells and the study of the cell cycle regulation of thymidylate synthase and dihydrofolate reductase. *Anal Biochem* **182**: 338–345

Mirjolet JF, Barberi-Heyob M, Marchal S, Colosetti P and Merlin JL (1997) In human cancer cell lines, thymidylate synthase expression and activity are principally associated with cells cycle variations. *Proc Am Assoc Cancer Res* **38**: 476

Pestalozzi BC, McGinn CJ, Kinsella TJ, Drake JC, Glennon MC, Allegra CJ and Johnston PG (1995) Increased thymidylate synthase protein levels are principally associated with proliferation but not cell cycle phase in asynchronous human cancer cells. *Br J Cancer* **71**: 1151–1157

Peters GJ and Van Groeningen CJ (1991) Clinical relevance of biochemical modulation of 5-fluorouracil. *Ann Oncol* **2**: 469–480

Peters GJ, Van Der Wilt CL and Van Groeningen CJ (1994) Predictive value of thymidylate synthase and dihydropyrimidine dehydrogenase. *Eur J Cancer* **30A**: 1408–1411

Plucinski TM, Fager RS and Reddy GP (1990) Allosteric interaction of components of the replitase complex is responsible for enzyme cross-inhibition. *Mol Pharmacol* **38**: 114–120

Riccardi A, Danova M, Wilson G, Ucci G, Dörmer P, Mazzini G, Brugnatelli S, Girino M, McNally NJ and Ascari E (1988) Cell kinetics in human malignancies studies with in vivo administration of bromodeoxyuridine and flow cytometry. *Cancer Res* **48**: 6238–6245

Schimke RT (1984) Gene amplification, drug resistance, and cancer. *Cancer Res* **44**: 1735–1742

Smith PK, Krohn RI, Hermanson GT, Mallia AK, Gartner FH, Provenzano MD, Fujimoto EK, Goeke NM, Olson BJ and Klenk DC (1985) Measurement of protein using bicinchoninic acid. *Anal Biochem* **150**: 76–85

Sommadossi JP, Gewirtz DA, Diasio RB, Aubert C, Cano JP and Goldman ID (1982) Rapid catabolism of 5-fluorouracil in freshly isolated rat hepatocytes as analyzed by high-performance liquid chromatography. *J Biol Chem* **4**: 2858–2864

Storms RK, Ord RW, Greenwood MT, Mirdamadi B, Chu FK and Belfort M (1984) Cell cycle dependent expression of thymidylate synthase in *Saccharomyces cerevisiae*. *Mol Cell Biol* **4**: 2858–2864

Takeishi K, Kaneda S, Ayusawa D, Shimizu K, Gotoh O and Seno T (1985) Nucleotide sequence of a functional cDNA for human thymidylate synthase. *Nucleic Acids Res* **13**: 2035–2043

© Cancer Research Campaign 1998